# Tryptophan Metabolic Pathways and Brain Serotonergic Activity: A Comparative Review

**DOI:** 10.3389/fendo.2019.00158

**Published:** 2019-04-08

**Authors:** Erik Höglund, Øyvind Øverli, Svante Winberg

**Affiliations:** ^1^Norwegian Institute of Water Research, Oslo, Norway; ^2^Centre of Coastal Research, University of Agder, Kristiansand, Norway; ^3^Department of Food Safety and Infection Biology, Faculty of Veterinary Medicine, Norwegian University of Life Sciences, Oslo, Norway; ^4^Behavioural Neuroendocrinology Group, Department of Neuroscience, Uppsala University, Uppsala, Sweden

**Keywords:** serotonin, stress, aggression, immune response, fatty acids, dietary supplementation

## Abstract

The essential amino acid L-tryptophan (Trp) is the precursor of the monoaminergic neurotransmitter serotonin (5-hydroxytryptamine, 5-HT). Numerous studies have shown that elevated dietary Trp has a suppressive effect on aggressive behavior and post-stress plasma cortisol concentrations in vertebrates, including teleosts. These effects are believed to be mediated by the brain serotonergic system, even though all mechanisms involved are not well understood. The rate of 5-HT biosynthesis is limited by Trp availability, but only in neurons of the hindbrain raphe area predominantly expressing the isoform TPH2 of the enzyme tryptophan hydroxylase (TPH). In the periphery as well as in brain areas expressing TPH1, 5-HT synthesis is probably not restricted by Trp availability. Moreover, there are factors affecting Trp influx to the brain. Among those are acute stress, which, in contrast to long-term stress, may result in an increase in brain Trp availability. The mechanisms behind this stress induced increase in brain Trp concentration are not fully understood but sympathetic activation is likely to play an important role. Studies in mammals show that only a minor fraction of Trp is utilized for 5-HT synthesis whereas a larger fraction of the Trp pool enters the kynurenic pathway. The first stage of this pathway is catalyzed by the hepatic enzyme tryptophan 2,3-dioxygenase (TDO) and the extrahepatic enzyme indoleamine 2,3-dioxygenase (IDO), enzymes that are induced by glucocorticoids and pro-inflammatory cytokines, respectively. Thus, chronic stress and infections can shunt available Trp toward the kynurenic pathway and thereby lower 5-HT synthesis. In accordance with this, dietary fatty acids affecting the pro-inflammatory cytokines has been suggested to affect metabolic fate of Trp. While TDO seems to be conserved by evolution in the vertebrate linage, earlier studies suggested that IDO was only present mammals. However, recent phylogenic studies show that IDO paralogues are present within the whole vertebrate linage, however, their involvement in the immune and stress reaction in teleost fishes remains to be investigated. In this review we summarize the results from previous studies on the effects of dietary Trp supplementation on behavior and neuroendocrinology, focusing on possible mechanisms involved in mediating these effects.

## Introduction

Tryptophan (Trp) is an essential amino acid in all animals, which is synthesized and provided to higher trophic levels by bacteria, fungi and plants. In addition to being a component for protein synthesis, Trp is also the obligatory substrate for the production of several important bioactive substances. For example, tryptophan is a substrate for the synthesis of serotonin (5-hydroxytryptpamine, 5-HT) in the brain and gut, and melatonin in the pineal gland. In vertebrates, central 5-HT plays an integrative role in the behavioral and neuroendocrine stress response (1–3). Accordingly, effects of dietary Trp on the neuroendocrine stress response have been reported in a variety of species, spanning from teleosts to humans (4–10). However, the mechanisms underlying this link between Trp metabolism and the stress response are not fully understood.

In mammals, the majority of Trp is catabolized and transformed through the kynurenic pathway to bioactive substances which potentially can interact with the stress response (11). Moreover, infections, stress, and changes in the gut microbiome have all been shown to shunt Trp metabolism from 5-HT production toward this pathway (12, 13). Consequently, pathological changes in stress responsiveness, as in depression, have been related to nutritional factors, stress and immune function in humans (14, 15). However, in non-mammals, information on the kynurenic pathway and its interactions with central 5-HT signaling and the stress response is scattered and/or limited.

Dietary manipulations affecting Trp availability to the brain have been used as a tool to investigate involvement of the 5-HT system in behavior, mood and cognition in humans (16–18). Likewise, the dietary Trp content have been shown to affect endocrine and behavioral responses to stress in teleost fishes (10, 19, 20). This review summarizes the results from previous studies on the effects of dietary Trp supplementation on the behavioral and neuroendocrine stress response, focusing on possible mechanisms involved in mediating these effects. We also present a hypothesis on how the diet could be used to improve fish stress tolerance through interactions with the Trp metabolic pathways.

## L-tryptophan Availability and Brain Serotonergic Activity

**Figure 1:**
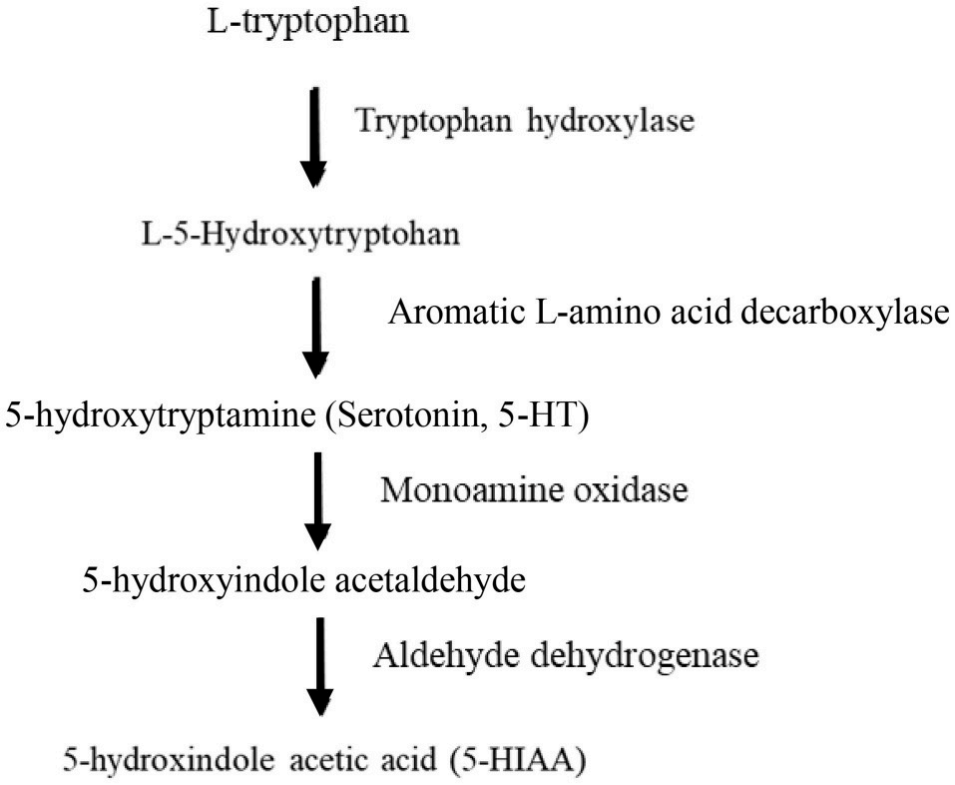
Biosynthetic pathway of serotonin.

In serotonergic neurons Trp serves as the precursor for 5-HT. The 5-HT metabolic pathway is initiated by Trp being hydroxylated to the intermediate 5-hydroxytryptophan (5-HTP), which is subsequently decarboxylated to become 5-HT. Tissue levels of 5-HTP are usually low since this substance is rapidly decaroxylated by the enzyme aromatic amino acid decarboxylase [for review see (21)]. Thus, the rate limiting step in the biosynthesis of 5-HT is the hydroxylation of Trp which is catalyzed by the enzyme tryptophan hydroxylase (TPH) (Figure 1). This enzyme is specific for 5-HT producing cells, however, it is present in two different isoforms, TPH1 and TPH2 [reviewed in (22, 23)].

In amniotes 5-HT neurons are only present in the raphe area of the hind brain whereas in anamniotes, including teleosts, 5-HT cell bodies are also located in pretectal areas and basal forebrain. In zebrafish (*Danio rerio*) raphe and pretectal 5-HT cells express TPH2, whereas diencephalic and hypothalamic 5-HT cells express TPH1 (TPH1a and TPH1b) and TPH3, respectively (23). Interestingly, TPH2 show a Km for its substrate which is in the range of *in vivo* brain levels of Trp (24). Consequently, the rate of 5-HT synthesis in cells expressing TPH2 is drastically affected by changes in Trp availability, an effect which is probably not seen in 5-HTergic cells expressing other TPH isoforms (22). Moreover, the rate of 5-HT synthesis is believed to be reflected in the release of 5-HT, often quantified as the concentration of the catabolite 5-hydroxyindole acetic acid (5-HIAA), or the 5-HIAA/5-HT ratio. Thus, changes in Trp availability may have direct effects on 5-HTergic tone. Coherent to this, Russo et al. (25) made the interesting suggestion that Trp may act as signal to the brain, transferring information on peripheral homeostasic challenges to the 5-HT system which in turn could act to defend homeostasis. Dietary composition as well as stress, physical activity and immune system activation will all have effects on plasma Trp concentrations, and thus on brain Trp availability and raphe 5-HTergic activity (25). Such Trp related changes in 5-HTergic activity could have direct effects on behavior as well as endocrine status through 5-HT projections to telencephalic and hypothalamic areas. It could be argued that such effects may be less important in teleost fish since they have extra-raphe located 5-HT cell populations expressing the TPH1 isoform, making them less responsive to changes in Trp availability. However, in teleosts, as well as in other vertebrates, the raphe 5-HTergic cells have a wide projection pattern innervating most brain regions (23). Still, it has to be acknowledged that very little is known about the role of telesost forebrain 5-HT cell population in the control of behavior and endocrine functions (23).

## Factors Affecting Trp Uptake to the Brain

### Dietary Effects on Trp Availability

**Figure 2:**
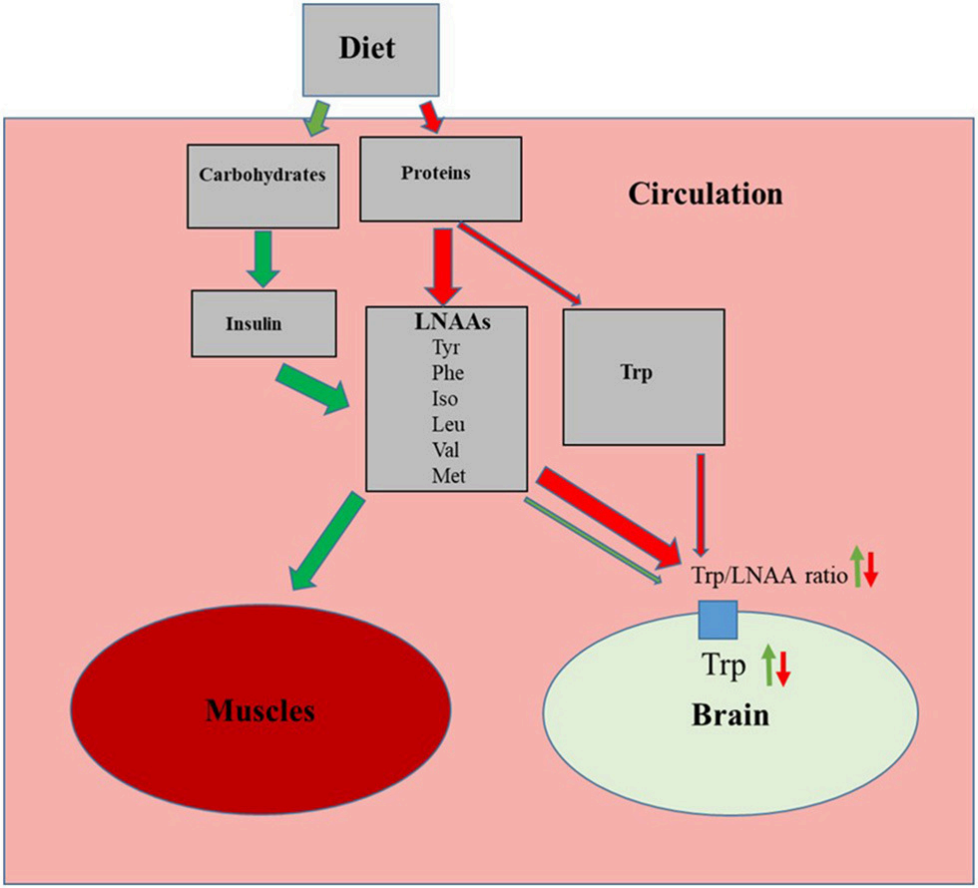
Effects of the proteins and carbohydrates on influx of tryptophan (Trp) to the brain. Green arrows indicate activation of carbohydrate induced pathway, resulting increased muscle uptake of large neutral amino acids (LNAAs; Tyr, tyrosine; Phe, phenylalanine; Iso, isoleucine; Leu, leucine; Val, valine and Met, methionine) which in turn increases plasma Trp/LNAA ratio and brain Trp levels. Red arrows indicate how a normal dietary protein source, with relatively low Trp content, decreases the plasma Trp/LNAA ratio and brain Trp levels.

The essential amino acid Trp enters the brain in competition with other large neutral amino acids (LNNAs; i.e., valine, isoleucine, leucine, tyrosine, phenylalanine and methionine) through a common transporter protein. Thus, the amount of Trp entering the brain depends on the plasma concentrations of Trp in relation to the other LNAAs [for references see reviews (26, 27)]. Hence, ingestion of a normal protein source, usually containing 0.5–1% Trp, results in a relatively small increase in Trp but a larger elevation of plasma concentrations of other LNNAs (28). This results in a decrease in the plasma Trp/LNAA ratio and thus reduced Trp influx to the brain (Figure 2). Dietary carbohydrates, on the contrary, increase brain Trp levels. This is due to elevated insulin which in turn promote uptake of LNAAs except Trp to the skeletal muscles, thereby increasing plasma Trp/LNAA ratio and Trp influx to the brain (Figure 2) (26, 27). This differential amino acid uptake to skeletal muscles is caused by the fact that Trp in blood plasma is bound to albumin whereas other LNAA are not. Trp influx to the brain is then promoted by the common LNAA transporter protein in the blood brain barrier having a much higher affinity for Trp compared to albumin (27).

Studies in rainbow trout (*Oncorhynchus myliss*) show that the amino acid composition of trout albumin differs from that of mammalians and lacks the binding site for indoles (29, 30). Thus, in rainbow trout, the majority of plasma Trp is in its free non-protein bound state (31, 32). This assumption is further strengthened by a study by Ruibal et al. (33) showing that hyperglycemia induced elevation of plasma insulin levels did not affect brain 5-HT activity in rainbow trout. It is not known if the lack of Trp binding by albumin is specific for rainbow trout or if it represents a more general trait of teleost albumin. However, it is possible that in teleost fishes brain influx of Trp could be more dependent of the dietary amino acid composition than on carbohydrates.

### The Kynurenic Pathway

**Figure 3:**
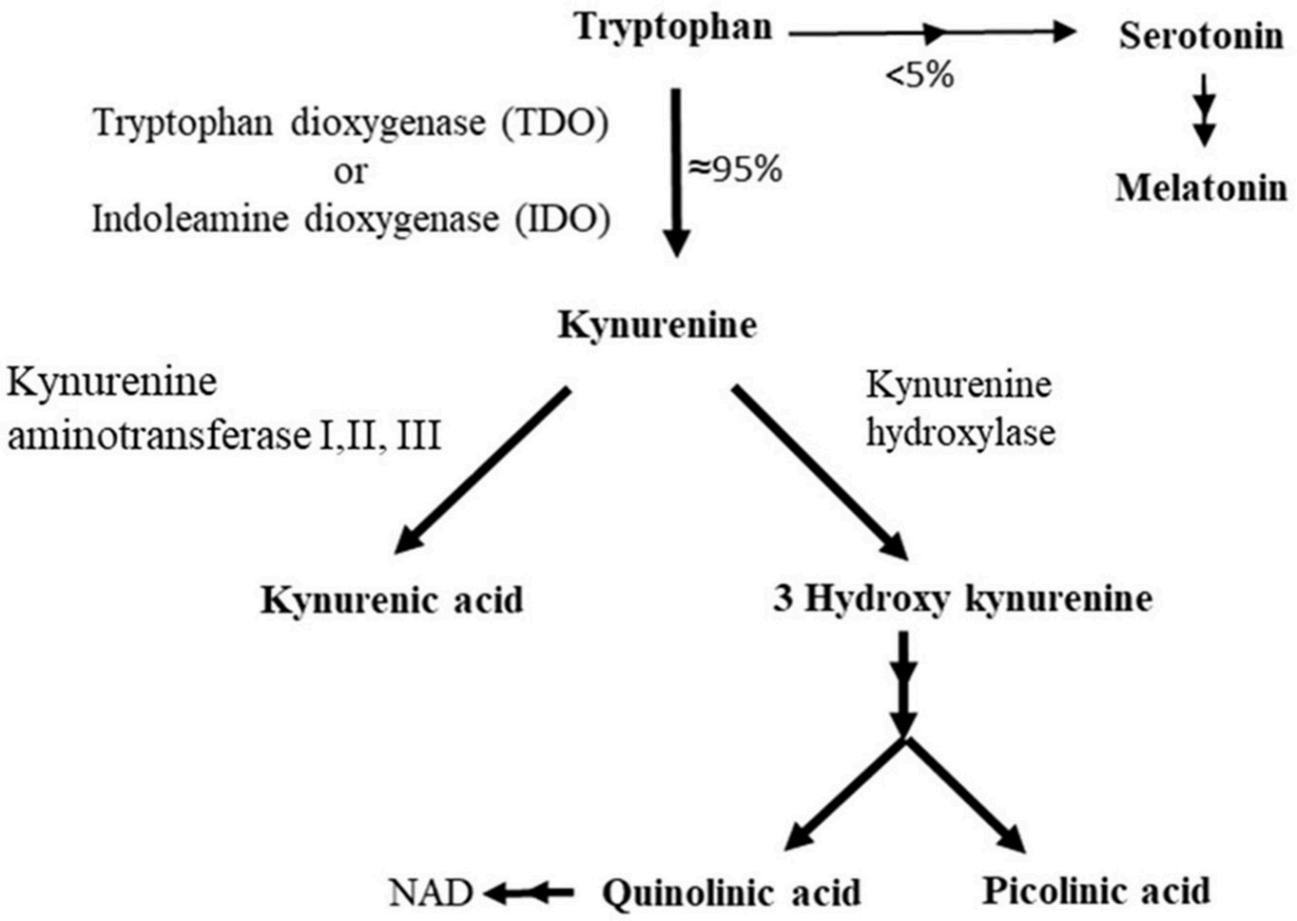
Major metabolic pathway of tryptophan in mammals.

In fact, only a minor fraction of the Trp pool is utilized for 5-HT biosynthesis. In mammals, the majority of Trp enters the kynurenic pathway and is converted to other bioactive substances than 5-HT, such as kynurenic acid and quinolinic acid (Figure 3) [for references see review (11)]. The first stage of this pathway is catalyzed by the hepatic enzyme tryptophan 2,3-dioxygenase (TDO) and the extrahepatic enzyme indoleamine 2,3-dioxygenase (IDO), enzymes that are induced by glucocorticoids and pro-inflammatory cytokines, respectively (34). Thus, chronic stress and infections can shunt available Trp toward the kynurenic pathway and thereby lowering brain 5-HT synthesis while simultaneously increasing the production of other Trp based bioactive substances. Moreover, since a majority of Trp follows the kynurenic pathway (<95%, Figure 3) relative small changes in the activity of this pathway can have rather big impact on the Trp influx to the brain (35). Accordingly, decreased Trp influx to the brain as a result of stress or inflammation/infection induced activation of the kynurenic pathway have been suggested to be an underlying factor for mental illnesses and dysregulation of the neuroendocrine stress axis (12, 14, 15).

Generally, IDO is more nonspecific than TDO, and catabolizes other indoleamines than Trp. Moreover, two distinct IDO genes, IDO1 and IDO2, have been identified in vertebrates. Earlier studies suggested that IDO1 arose by a gene duplication in mammals (36). However, recent phylogenetic analyses show that IDO1 are present in reptiles and in teleosts, indicating that the gene duplication occurred in the common ancestor of vertebrates (37). In mammals, the activation of dendritic cells results in IDO1 induction with the depletion of Trp levels locally or systemically, a mechanism by which interferons inhibit the growth of certain bacteria, intracellular parasites, and viruses (34). Moreover, an elevation of the activity of the kynurenic pathway also inhibits T lymphocyte replication which results in immunosuppression and tolerogenicity. In line with this, IDO1 have been suggested to play an important role in preventing fetal rejection and in facilitating immune escape of tumor cells (34). In addition, some products of the kynrunic pathway may act anti-inflammatory (38, 39). However, to which extent these anti-inflammatory Trp catabolites acts back on the activity kynurenic pathway and thereby affecting Trp influx to the brain and/or central 5-HT signaling is to our knowledge unknown.

The Trp catabolizing efficiency of IDO2 and non-mammalian IDO1 seems to be lower than mammalian IDO1, and their function and involvement in the immune response in comparative model species is far less understood (37). However, recently, it has been demonstrated that treatment with bacterial lipopolysaccharide (LPS) induces and upregulation of IDO expression in rainbow trout, suggesting that this enzyme is involved in the immune response in non-mammalian vertebrates (40). Moreover, in the aforementioned study, expression of IDO was induced by the pro-inflammatory cytokine interferon gamma (IFNγ) in an *in vitro* cell model, indicating similar induction mechanisms as those in mammalian IDO1 (40). This suggests that systemic infection may decrease Trp influx to the brain of teleost fishes in the same way as in mammals, and result in behavioral and physiological changes (see section Kynurenine pathway).

### Acute Stress

As discussed above chronic stress may result in lowered brain Trp availability as a consequence of a stress-induced activation of the kynurenine pathway. However, acute stress has been reported to have the opposite effect elevating brain Trp levels in both mammals (41, 42) and teleost fish (3, 10). This stress-induced increase in brain Trp concentrations appears at least in part related to a sympathetic activation and elevated levels of circulating plasma catecholamines (43). Plasma catecholamines stimulate lipolysis, resulting in elevated plasma levels of non-esterified fatty acids, which in turn could compete with Trp for binding to albumin and thus elevate the plasma pool of free Trp available for uptake into the brain [reviewed by (44)]. However, as discussed above, rainbow trout albumin appears to lack the Trp binding site, suggesting that mechanisms based on competition between Trp and non-esterified fatty acids are not involved in stress-induced increase in brain Trp in teleosts, at least not in rainbow trout. It has also been suggested that sympathetic activation results in increased permeability of the blood-brain barrier, another mechanism that could increase brain Trp influx (44).

## Trp and the Neuroendocrine Stress Response

### Stress Responses Are Modified by Trp Availability and Brain 5-HT Functions

As mentioned earlier in this review, the positive relationship between Trp availability and brain 5-HT production is well conserved within the vertebrate linage. Coherent to this, the involvement of 5-HT in the neuroendocrine regulation of the stress response seems to be similar within this linage. 5-HT plays a central role in control of the hypothalamus–pituitary–adrenal axis (HPA axis) in mammals, and the hypothalamic–pituitary–interrenal axis (HPI axis) in fish. This, mainly through its effects on the release of corticotropin-releasing factor (CRF) from the hypothalamus (45, 46). In addition, extra hypothalamic 5-HT appears be involved in appraisal and stress coping mechanisms, modulating behavioral and neuroendocrine responses to stressors (47, 48). Furthermore, as mentioned in section The Kynurenic pathway and Acute stress, stress by itself can influence the Trp influx to the brain, and thereby affect 5-HT signaling and the stress response. Moreover, the HPA/HPI axis are under feedback control on serval levels, including central 5-HT signaling. Thus, the link between Trp and the 5-HT system and how they control behavioral and neuroendocrine stress responses appears complex with 5-HT having context dependent effects (19, 22, 49).

### Effects of Elevated Dietary Trp

Long-term effects of Trp dietary manipulations on the neuroendocrine stress response have been observed in both mammals and teleost fishes [for a review see (49)]. For instance, in pigs, elevated dietary Trp had stress suppressive effects, including elevated hypothalamic 5-HT and lowered post stress plasma cortisol levels, effects that peaked after 5 days of dietary Trp enrichment (50). Similarly, (51) showed that post-stress plasma cortisol levels returned to baseline earlier after social stress in pigs fed Trp enriched feed for 7 days. Interestingly, a similar time frame for the suppressive effects of dietary Trp supplementation on glucocorticoid release has also been demonstrated in fish (for references see Table 1). For instance, studies in rainbow trout show that suppression of the neuroendocrine stress response is present after 7, but not after 3 or 28 days of treatment with dietary Trp supplementation (52). Furthermore, in the earlier studies showing a suppressive effect of elevated dietary Trp on the neuroendocrine response to an acute stressor the effects were investigated during or directly following a period of dietary Trp supplementation (10, 52). However, in recent studies in sea water reared Atlantic salmon (*Salmo salar*), the suppressive effect on post-stress plasma cortisol seems to appear between 2 and 8 days after terminating the Trp supplementation. Moreover, in Atlantic salmon, this suppressive effect was still present at 21 days post Trp supplementation (7, 53). Basic et al. (53) suggested that such slow acting Trp-induced alterations of HPI-axis reactivity could be related to smoltification, a process where salmonid fish adapt to sea water. Moreover, these long-term alternations of HPI axis reactivity was not related to changes in hypothalamic 5-HT neurochemistry. Instead they coincided with changes in dopaminergic neurochemistry in this brain part, effects which may be related to elevated activity of the kynruneric pathway, as discussed in section The Kynurenic pathway. Similar results were shown in the study performed by Höglund et al. (7), where 5-HTergic activity in hypothalamus did not follow the long term Trp induced suppressive effect on post stress cortisol levels. The latter study also included telencephalon and 5-HT activity followed the same general pattern as cortisol in this brain part. Höglund et al. (7) suggested that such region specific differences could be related to 5-HT signaling in telencephalon being more dependent on projections from the hindbrain raphe, a nucleus where 5-HT neurons are highly sensitive to available Trp, see section L-tryptophan availability and brain serotonergic activity.

**Table 1:** Effects of dietary tryptophan supplementation on the behavioral and endocrine stress response in teleost fishes.

**Species**	**Dose (x std feed)**	**Treatment (days)**	**Behavior**	**Plasma cortisol**		**Stressor**		**References**
				**Baseline**	**Stress**	**Type**	**Post Trp terat. (days)**	
*Oncorhuncus mykiss*	2	7	N. i.	–	–	Confinement 2 h	1	(10)
	4	7	N. i.	↑	↓	Confinement 2 h	1	
	8	7	N. i.	↑	↓	Confinement 2 h	1	
*Oncorhuncus mykiss*	8	3	N. i.	↑	-	Confinement 2 h	1	(52)
	8	7	N. i.	–	↓	Confinement 2 h	1	
	8	28	N. i.	–	–	Confinement 2 h	1	
*Gadus morhua*	2	7	N. i.	–	–	Confinement (0.5 h)	1	(4)
	2	7	N. i.	–	–	Confinement (0.5 h)	2	
	2	7	N. i.	–	–	Confinement (0.5 h)	4	
	3	7	N. i.	–	–	Confinement (0.5 h)	1	
	3	7	N. i.	–	–	Confinement (0.5 h)	2	
	3	7	N. i.	–	–	Confinement (0.5 h)	4	
	4	7	N. i.	–	↓	Confinement (0.5 h)	1	
	4	7	N. i.	–	–	Confinement (0.5 h)	2	
	4	7	N. i.	–	–	Confinement (0.5 h)	4	
*Salmo salar*	2	7	N. i.	–	–	Confinement (0.5 h)	1	(53)
	2	7	N. i.	–	–	Confinement (0.5 h)	2	
	2	7	N. i.	↓	↓	Confinement (0.5 h)	10	
	3	7	N. i.	↓	–	Confinement (0.5 h)	1	
	3	7	N. i.	–	–	Confinement (0.5 h)	2	
	3	7	N. i.	↓	↓	Confinement (0.5 h)	10	
	4	7	N. i.	–	–	Confinement (0.5 h)	1	
	4	7	N. i.	–	↑	Confinement (0.5 h)	2	
	4	7	N. i.	↓	↓	Confinement (0.5 h)	10	
*Oreochromis niloticus*	4	7	N. i.	–	–	Chasing (0.3 h)	0	(54)
	10	7	N. i.	↓	–	Chasing (0.3 h)	0	
*Salmo salar*	2	7	N. i.	–	–	Crowding (1 h)	8	(7)
	2	7	N. i.	–	↓	Crowding (1 h)	21	
	3	7	N. i.	–	–	Crowding (1 h)	8	
	3	7	N. i.	–	↓	Crowding (1 h)	21	
*Dicentrarchus labrax*	2	14	N.i	–	↑	24 h post immune challenge	0	
*Cyprinus carpio*	5	15	N. i.	N. i.	↓	Saltwater (6 h)	0	(55)
*Cyprinus carpio*		21	N. i.	↓	↓	Cu+ exsposure (7 days)	0	(56)
*Cichlasoma dimerus*	8	28	N. i.	↓	N. i.			(57)
*Labeo rohita*	1.7^a^	45	N. i.	↓	N. i.			(58)
	2.4^a^	45	N. i.	↓	N. i.			
	2.9^a^	45	N. i.	↓	N. i.			
*Labeo rohita*	2.8	60	N. i.	N. i.	↓	Temp and/or salt (30 days)	0	(59)
	4.8			N. i.	↓	Temp and/or salt (30 days)	0	
*Cirrhinus mrigala*	~3^a^	60	N. i.	↓	↓	High rearing density (60 days)	0	(60)
*Sander lucioperca*	3	7-60	N. i.	N. i.	↓	Emersion	0	(61)
	6	7-60	N. i.	N. i.	↓	Emersion	0	
**AGGRESSION**								
Oncorhyncus mykiss	36	0	–				0	(20)
		3	–				0	
		7	↓				0	
	360	0	–				0	
		3	–				0	
		7	↓				0	
*Oncorhuncus mykiss*	8	7	↓	N. i	↓	A smaller conspefic (1 h)	1	(62)
*Gadus morhua*	6^a^	4–10	↓	N. i	N. i	3 × social interact. (0.15 h/day)	0	(63)
*Matrinxã Brycon amaz*.	2	7	↓	N. i.	↓	Social interaction (0.3 h)	0	(64)
	4	7	↓	N. i.	↓	Social interaction (0.3 h)	0	
			Anneroxia					
*Salmo trutta*	3.6	7	↓	N. i.	N. i.	Novel environment (3 days)	1–3	(19)

*↑, ↓, and – refers to stimulating, suppressive or no effect t compared to standard feed*.

*N.i. Not investigated*.

a *Estimated from similar feed recipe*.

Generally, teleost fishes have a remarkable neurogenic and regenerative capacity throughout ontogeny, and it has been suggested that structural changes may underlie long-lasting effects on telencephalic neurochemistry induced by elevated dietary Trp in teleost fishes (7). This type of brain architectural changes is supported by mammalian studies, showing that the 5-HT system is involved in the organization and development of its own neural projection pattern (65). In addition, a positive relationship between dietary Trp content and neural proliferation markers, such as (exogenous) 5-bromo-2-deoxyuridine and brain derived neurotrophic factor (BDNF) has been demonstrated in rats (66), which lends further support for the suggestion that dietary Trp can induce structural changes in the brain.

There are studies in teleost fishes showing effect of longer Trp treatment periods than 7 days (Table 1). For example, Tejpal et al. (60) showed that a 60 days of dietary Trp supplementation decreased baseline plasma cortisol values as well as the cortisol response to 60 days of crowding stress. Moreover, longer Trp treatment periods have also been shown to act stimulatory on plasma cortisol responses. For example, an immune challenge by i.p. injection of inactivated *Photobacterium damselae* suspension resulted in elevated cortisol values in seabass fed Trp supplemented feed for 2 weeks as compared to fish given standard feed fish (67). Furthermore, there is a rather high variability in the effect of elevated dietary Trp on baseline cortisol values (Table 1). This variability could reflect interspecific differences in Trp metabolism and neuroendocrine mechanisms (49). Moreover, Höglund et al. (19) suggested that such variation could be related to differences in HPI axis activation due to divergent rearing environments. For example, in the studies performed by Lepage et al. (10, 52, 62), fish were kept socially isolated while in other studies they were group reared (4, 7, 53, 54). Considering the fact that the 5-HT system is affected by social interaction (3, 22, 68), this type of rearing differences may explain some of the variability in the response to elevated dietary Trp. Moreover, studies in humans and rats suggest that individual variation in 5-HT neurotransmission underlies differences in the response to dietary Trp manipulation (27). It has become increasingly clear that individual variation in HPA/I axis reactivity is as widespread phenomena in the vertebrate linage (69). Still, if such individual variation is related to sensibility to dietary manipulations of dietary Trp content in non-mammalian vertebrates remains to be investigated.

### Kynurenine Pathway

As mentioned above, in the section about factors affecting Trp uptake to the brain. Trp influx to the brain and brain 5-HT signaling can be modulated by the activation of the kynurenic pathway. In addition, metabolites of this pathway may affect neuronal signaling involved in stress coping processes [reviwed by (14)]. The metabolite in the first step of this pathway, kynurenine, readily passes the blood brain barrier (70). In the brain it is further degraded to kynurenic acid or quinolinic acid. Further down this pathway quinolinic acid produces neurotoxic compounds such as NMDA receptor agonists and oxidative radicals (71) while kynurenic acid is neuroprotective by being an NMDA receptor antagonist [for references see (14)]. In mammals, the neuroprotective kynurenic acid is mainly produced in astrocytes, while neurotoxic compounds are produced in macrophages and microglia (34). It has been suggested that an imbalance between these neurodegenerative and neuroprotective factors are involved in brain dysfunctions, including poor stress coping ability, in depression (72). In addition, studies in rats show that dietary Trp can affect brain levels of kynurenic acid (73), which in turn effects other neurotransmitters, such as dopamine and glutamine through activation of NMDA and/or a7 nicotinic acetylcholine receptor (74, 75). Central effects of Trp metabolites produced by the kynurenic pathway in teleost fishes are, to our knowledge, largely unknown. Still, effects of dietary Trp supplementation on dopaminergic neurochemistry in Atlanitic salmon (53) and Atlanitic cod (*Gadus morhua*) (4) have been suggested to be related to elevated levels of kynurenic acid (53).

## Behavioral Effects of Elevated Dietary Trp

There is a general consensus that low levels of central 5-HT are associated with high levels of aggression within the vertebrate subphylum (3, 69). In line with this, human studies show that alterations of the dietary Trp content changes irritability and aggressive behavior [for references see review by Young and Leyton (76)]. For example, human lab studies show that dietary Trp induces a dose dependent effect on aggressive responses, where Trp supplementation and depletion induced the lowest highest aggression, respectively (77, 78). This negative relationship between dietary Trp content and aggression is further supported by studies on rats and birds, showing that Trp loading can attenuate aggressiveness (79, 80). Similarly, there are studies in teleost fishes showing a general suppressive effect on aggressive behavior by dietary Trp supplementation (20, 63, 64). Furthermore, in the study performed by Winberg et al. (20) the attenuating effects of dietary Trp on aggressive responses during territorial defense followed the same time-coarse as the effects on the neuroendocrine stress response in rainbow trout (52), with a peak after 7 days of treatment. This together with a study performed by Höglund et al. (19), showing that the same treatment time attenuated the anorexic response to a novel environment, strongly suggest that Trp affects 5-HT signaling and the integrating role of this neurotransmitter in behavioral and neuroendocrine stress responses.

Dietary Trp supplementation have also been shown to reduce cannibalism in juvenile grouper (*Epinephelus coioides*) (81) and pike perch (*Sander lucioperca*) (82). However, the behavioral components of this response were not studied. Differences in body size is a main factor underlying cannibalism in piscivorous fish (83), and one possible explanation to the reduced cannibalism could be a more homogeneous growth due to reduced competition for food in fish given Trp supplemented food. The behavioral effect of dietary Trp manipulations in teleost fishes are summarized in Table 1.

## Conclusions and Suggestion for Direction of Further Studies

A positive relationship between dietary Trp and brain 5-HT activity seems to be present across the vertebrate linage. However, there appear to be differences between teleost fishes and mammals when it comes to plasma Trp transport since teleost albumin lacks the indole binding site (29, 30). This makes Trp influx to the brain less sensitive to carbohydrates in fish compared to mammals. On the other hand, behavioral and neuroendocrine effects of elevated dietary Trp are similar in all vertebrates. Studies in mammals and teleost fishes show that these effects, including suppression of aggressive behavior, attenuation of stress induced anorexia and lower post stress plasma cortisol, appear after 3–7 days of elevated dietary Trp intake. It has been suggested this slow time-course reflects 5-HT induced structural changes in the brain (7). However, further studies are needed to verify this assumption.

In mammals the majority of Trp enters the kynurenic pathway. The first stage of this pathway is catalyzed by the enzymes TDO and IDO that are induced by glucocorticoids and pro-inflammatory cytokines, respectively. Thus, chronic stress and infections can shunt available Trp toward the kynurenic pathway and thereby lowering the rate of brain 5-HT synthesis while simultaneously increasing the production of other Trp metabolites [for references see (14)], which potentially can affect behavioral and endocrine responses to stress. So far, the kynurenic pathway have been neglected when investigating effects of dietary Trp supplementation in teleost fishes. It has previously been pointed out that effects of dietary Trp is context dependent, where especially the stress status of the animals can affect the outcome of dietary Trp manipulation (19). A recent study demonstrates that the expression of IDO mRNA is upregulated by LPS in rainbow trout (40), suggesting that bacterial infection can affect the catabolic faith of Trp also in fish. Previously dietary Trp supplementation have been suggested as a strategy for reducing unavoidable stress, such as stress related to transport, size grading and vaccination, in aquaculture (84). However, considering that inflammatory processes might affect the catabolic faith of Trp in teleost fish, anti-inflammatory treatments should also be considered.

In humans, low circulating levels of the ω3 fatty acids, eicosapentaenoic acid (EPA) and docosahexaenoic acid (DHA), and a decreased ratio of EPA to the ω6 fatty acid arachidonic acid (ARA) have been associated with psychiatric ailments and poor stress coping ability (15). Moreover, a diet with high DHA and EPA have been shown to affect serotonergic transmission and to prevent such psychiatric ailments [for references see (15)]. The mechanisms for this anti-depressive action of ω3 fatty acids are currently not fully understood. However, it is possible that a diet with high ω3 content results in a suppression of pro-inflammatory eicosanoids, which in turn may reduce the activity of the kynurenic pathway, increasing Trp influx to the brain, and subsequently stimulate brain 5-HT synthesis.

The relative amount of marine ω3 fatty acids has decreased in commercial fish feed. Potentially, this may result in poorer stress coping ability trough dietary effects on central 5-HT signaling. Thus, we hypothesize that it is not only the relative amount of Trp to other LNAAs in the diet that is important for producing stress resilient robust fish. Rather, there is an interplay between dietary amino and fatty acids that decides the effects of Trp supplementation, where ratio ω3 to ω6 fatty acids in the diet influences the catabolic faith of Trp. Studies demonstrating a negative relationship between HPI-axis reactivity and the ration of ω3 to ω6 fatty acids in the diet (85, 86) lends support to this hypothesis. However, if such effects of dietary fatty acid composition are related to changes in the activity of the kynurenic pathway is currently not known.

## Author Contributions

EH and SW drafted the manuscript. EH, ØØ, and SW finalized the manuscript.

### Conflict of Interest Statement

The authors declare that the research was conducted in the absence of any commercial or financial relationships that could be construed as a potential conflict of interest.
